# Predictors of Standard Follow-Up Completion after Sexual Exposure to HIV: Five-Year Retrospective Analysis in a French HIV-Infection Care Center

**DOI:** 10.1371/journal.pone.0145440

**Published:** 2015-12-22

**Authors:** Pierre Gantner, Michele Treger, Constance De Miscault, Marie-Laure Batard, Claudine Bernard-Henry, Christine Cheneau, Erik De Mautort, Marialuisa Partisani, Michele Priester, David Rey

**Affiliations:** 1 Le Trait d’Union, HIV-infection care center, Hôpitaux Universitaires de Strasbourg, Strasbourg, France; 2 Biostatistics Laboratory, Faculté de Médecine, Université de Strasbourg, Strasbourg, France; Fudan University, CHINA

## Abstract

**Objectives:**

The care of exposed individuals to HIV remains a challenge regarding follow-up completion and HIV-testing of the partner. Identifying patients with risk of not fulfilling HIV-testing follow-up completion (FC), among patients demanding non-occupational post-exposure prophylaxis (nPEP), may improve clinical practice.

**Methods:**

A retrospective chart review was conducted in a single French HIV-infection care center. FC predictors were assessed in a multivariate logistic regression model (Likelihood ratios test).

**Results:**

Between 2009 and 2013, 646 sexual exposures to HIV were evaluated for nPEP, of which 507 effectively received nPEP (78%). FC rate was 30% (194/646). In the multivariate analysis, FC rates rose with age of exposed individuals (OR, 1.04 [0.25–4.28]; p<0.001) and decreased with the year of sexual exposure (OR, 0.74 [0.65–0.85]; p<0.001). FC was associated with sexual encounter with a sex worker (OR, 4.07 [0.98–16.82]; p<0.001) and nPEP use (OR, 2.69 [2.37–3.06]; p<0.001). nPEP early discontinuation was associated with decreased FC rates (OR, 0.18 [0.08–0.39]; p<0.001). No documented nPEP failure was identified. However, five Men who have Sex with Men (MSM) nPEP recipients for unprotected anal receptive intercourse subsequently seroconverted to HIV more than 6 months after nPEP. Seroconversion to HIV was associated with the lack of FC (p = 0.04) and multiple presentations for nPEP over the study period (p = 0.002).

**Conclusions:**

We identified significant predictors of not fulfilling sequential HIV-testing. They appear to be linked with a self-perceived HIV risk, especially in young adults recently exposed. Enhanced counseling in targeted individuals with high risk behaviors and using smartphone and internet-based strategies may be interesting retention in care options.

## Introduction

Biomedical prevention care for sexual exposure to HIV is recommended in both the US and Europe [1–2]. Non-occupational post-exposure prophylaxis (nPEP), a 28-day course of usually three antiretroviral drugs, should be discussed with all sexual exposures to HIV weighing up the risk of HIV transmission and the potential for harm due to nPEP [3–4]. Although nPEP is used since the late 1990s [5], comprehensive data that address current sexual exposures to HIV care practices are limited [6–9], suggesting that it is often underused by men who have sex with men (MSM) [6]. Besides 8 to 20% of patients stop nPEP due to adverse events [10–13]. Recently, high-risk sexual behavior [14], not having a partner known to be HIV-infected and prescription of a three-drug regimen [15] have been associated with decreased adherence.

National Recommended standard follow-up includes HIV antibodies testing at baseline and six weeks; or at baseline, eight and 16 weeks after sexual exposure for untreated and treated patients, respectively [16]. Among patients demanding nPEP, 30–38% are documented to have completed the HIV-testing follow-up [17–18]. Targeting patients with risk of not fulfilling HIV-testing follow-up completion (FC) may improve clinical practice.

The primary objective of this study was to identify predictors of HIV-testing FC among patients demanding nPEP at the University Hospital of Strasbourg, France. Secondary objectives were to determine trends in the sexual exposures, condom use and to describe our nPEP experience.

## Methods

### Setting and study period

We conducted a retrospective electronic medical files review of patients evaluated after sexual exposure to HIV between January 2009 and December 2013, at our institution. The local Ethics Committee of the Medicine Faculty, Strasbourg, France, approved the study. All participants gave prospective written consent for their clinical data to be anonymized and then analyzed for research purposes (Commission Nationale de l’Informatique et des Libertés number: 1193778).

### Sexual exposures

The following variables were collected: age, gender, condom use, relationship to the sexual partner, HIV status of sexual partner, sexual preference, sexual intercourse (anal receptive or insertive, vaginal receptive or insertive, oral receptive or insertive), factors increasing transmission risk, time from exposure to baseline visit at our center, returning for more sexual exposures over the study period and seroconversion at the end of the study (2014).

### Follow-up and nPEP

Since January 2000, the policy at our institution has been to prescribe lamivudine/zidovudine (150/300 mg twice a day) for 28 days, plus nevirapine (NVP) for the first 4 days only, at a dose of 200 mg once daily. We believe that such a short course of NVP is safe, supported by its long half-life, the lack of skin toxicity and a low incidence of liver toxicity, as previously published [19–20]. A four-day starter nPEP kit, including the whole NVP course, is available either in our HIV department, or in the emergency unit. A physician from the HIV-infection care center or the emergency unit first assesses the risk of HIV transmission. The follow-up is done at the HIV-infection care center, reference for sexual exposure to HIV care at our institution. Hematologic and hepatic evaluations are performed during follow-up.

nPEP discontinuation before treatment completion was collected. FC after a sexual exposure to HIV evaluation was defined by an HIV-testing (4^th^ generation ELISA combined antigen-antibody laboratory assay) performed at D0, W8 and W16 or D0 and W6 for patients receiving nPEP or not, respectively.

### Statistical Analysis

Chi-squared test, Kruskal-Wallis rank sum test and Fisher's exact test were conducted to identify any significant change over the study period.

To assess characteristics of patients with and without FC, non-parametric tests were used for univariate analyses (Wilcoxon and Fisher’s exact tests). Variables achieving a *p* value <0.17 were entered into a multivariate logistic regression model with a backward stepwise method based on the likelihood ratio test. Regarding repeated exposures during the study period, mixed effects logistic regression to model binary outcome variables, in which the log odds of the outcomes are modeled as a linear combination of the predictor variables, were used to fit a generalized linear mixed model. Seroconversions data were assessed by patient with Fisher’s exact test.

Statistical analyses were performed with R software (R Foundation, Vienna, Austria). Odd-ratios (OR) are given with a 95% confidence interval (CI).

## Results

### Characteristics of the study population

During the study period, 646 sexual exposures to HIV of 602 patients were evaluated for nPEP, of which 507 effectively received nPEP (78%) and 139 did not receive nPEP (22%) because of lack of indication to the provider (70%) and presentation >48 hours after sexual exposure (30%). Among the 602 patients, 6% (n = 37) presented for several exposures (range, 2–3). The cohort was composed of women (30%), heterosexual men (36%) and MSM (34%). Condomless sex was reported in 45% of intercourses (Table 1). The majority of exposures were consensual (90%). nPEP was provided to 93% of sexual assault. The HIV serostatus of the sexual partner remained unknown in 516 cases (83%), among them, only 46 (9%) could be tested through the study (all negative). Median time delay from sexual exposure to arrival to HIV-infection care center (n = 367) was 22 hours (IQR, 12–43).

**Table 1:** Trends in sexual exposures’ characteristics among five years. (n = 646). FC, Follow-up Completion; IQR, Interquartile Range; MSM, Men who have Sex with Men; nPEP, Non-Occupational Post-Exposure Prophylaxis.

	Total (n = 646)	2009 (n = 115)	2010 (n = 126)	2011 (n = 139)	2012 (n = 128)	2013 (n = 138)	p
Gender, n (%)							0.01
Female	193 (30%)	31 (27%)	48 (38%)	35 (25%)	48 (37%)	31 (22%)	
Male	453 (70%)	84 (73%)	78 (62%)	104 (75%)	80 (63%)	107 (78%)	
Median age, years (IQR)	29 (23–36)	27 (23–35)	28 (23–37)	29 (24–36)	31 (23–37)	29 (24–37)	0.6
Sexual type, n (%)							0.2
Heterosexuals	428 (66%)	74 (64%)	88 (70%)	81 (58%)	91 (71%)	94 (68%)	
MSM	218 (34%)	41 (36%)	38 (30%)	58 (42%)	37 (29%)	44 (32%)	
Sexual partner, n (%)							<0.0001
Casual	512 (81%)	93 (81%)	111 (92%)	107 (78%)	97 (76%)	104 (78%)	
Regular	71 (11%)	22 (19%)	9 (7%)	14 (10%)	15 (12%)	11 (8%)	
Multiple	6 (1%)	0	1 (1%)	2 (2%)	2 (2%)	1 (1%)	
Unknown	45 (7%)	0	0	14 (10%)	13 (10%)	18 (13%)	
Partner’s HIV status, n (%)							0.04
Unknown	512 (83%)	86 (83%)	105 (85%)	105 (80%)	106 (85%)	110 (83%)	
Negative	16 (3%)	6 (5%)	2 (2%)	4 (3%)	2 (2%)	2 (1%)	
Positive	100 (16%)	23 (22%)	16 (13%)	22 (17%)	16 (13%)	23 (16%)	
First nPEP evaluation, n (%)							0.3
Emergency department	246 (40%)	34	54	60	47	51	
HIV infection care center	367 (60%)	71	68	74	72	82	
Median delay between sexual exposure and nPEP evaluation at HIV infection care center, hours (IQR)	22 (12–43)	19 (12–38)	28 (15–53)	17 (11–43)	33 (13–65)	21 (12–41)	0.007
Exposure type, n (%)							
Vaginal Insertive	214 (34%)	36 (32%)	34 (27%)	46 (34%)	42 (33%)	56 (42%)	0.1
Vaginal Receptive	177 (28%)	30 (27%)	44 (35%)	33 (24%)	44 (35%)	26 (20%)	0.02
Anal Insertive	126 (20%)	19 (17%)	29 (23%)	36 (26%)	19 (15%)	25 (18%)	0.1
Anal Receptive	117 (18%)	26 (23%)	24 (19%)	23 (17%)	25 (20%)	19 (14%)	0.5
Oral Insertive	86 (14%)	23 (20%)	21 (17%)	19 (14%)	10 (8%)	13 (10%)	0.03
Oral Receptive	107 (17%)	25 (22%)	17 (13%)	25 (19%)	17 (13%)	23 (17%)	0.3
Condom use, n (%)							0.02
Yes	37 (6%)	15 (13%)	8 (6%)	6 (4%)	2 (2%)	6 (5%)	
Slip/Broke	311 (49%)	45 (40%)	61 (46%)	75 (56%)	61 (48%)	69 (51%)	
No	285 (45%)	53 (47%)	57 (45%)	54 (40%)	63 (50%)	58 (44%)	
Special condition, n (%)							
Intercourse with a sex worker	100 (16%)	21 (20%)	13 (11%)	24 (18%)	15 (12%)	27 (20%)	0.6
Rape	69 (11%)	9 (9%)	22 (18%)	11 (8%)	19 (15%)	8 (6%)	0.01
Anal/vaginal ejaculation	312 (51%)	62 (60%)	64 (52%)	52 (39%)	65 (52%)	69 (52%)	0.2
Blood at the site of exposure	67 (10%)	14 (13%)	18 (15%)	14 (11%)	8 (6%)	13 (10%)	0.4
nPEP provided, n (%)	507 (78%)	76 (66%)	108 (86%)	115 (83%)	98 (77%)	110 (80%)	0.004
Stopping nPEP before 28 days of treatment, n (%)	70 (18%)	8 (13%)	14 (17%)	15 (17%)	15 (18%)	18 (24%)	0.6
FC, n (%)	194 (30%)	41 (36%)	49 (39%)	39 (28%)	40 (31%)	25 (18%)	0.003

### HIV-testing follow-up

No patients were tested positive for HIV at baseline, and no documented nPEP failure was identified. However, five MSM (of 217 MSM, 2%) nPEP recipients for unprotected anal receptive intercourse subsequently seroconverted to HIV, all more than 6 months after nPEP. Over the study period, none of the five MSM, totalizing 10 nPEP prescriptions, achieved FC. Seroconversion to HIV was associated with the lack of FC (FC rate, 0/10 VS 194/442; p = 0.04). Three of five presented more than once for nPEP evaluation. Among patients consulting for consensual MSM exposures (n = 217), multiple presentations for nPEP over the study period was associated with subsequent seroconversion (3/13 VS 2/204; p = 0.001).

FC rate was 30% (n = 194). In the multivariate analysis (Table 2), FC rose with the age of exposed individuals (OR increase per year of age, 1.04 [0.25–4.28]; p<0.001) and there is a trend with decreasing rates of FC over time (OR decrease per year of sexual exposure, 0.74 [0.65–0.85]; p<0.001). FC was associated with sexual encounter with a sex worker (OR, 4.07 [0.98–16.82]; p<0.001) and nPEP use (OR, 2.69 [2.37–3.06]; p<0.001). nPEP early discontinuation was associated with decreased FC rates (OR, 0.18 [0.08–0.39]; p<0.001).

**Table 2:** Significant predictors associated with follow-up completion (FC) and condom use. CI, Confidence Interval; FC, Follow-up Completion; IQR, Interquartile Range; nPEP, Non-Occupational Post-Exposure Prophylaxis; NS, Not Significant; OR, Odd-Ratio. a, per year; b, per hour.

	**Follow-up completion (n = 646)**		**Univariate analysis (p)**	**Multivariate analysis (p)**	**OR (95% CI)**
	**Yes (n = 194)**	**No (n = 452)**			
Year of sexual exposure, year (IQR)	2011 (2010–2012)	2011 (2010–2013)	<0.001	<0.001	0.74^a^ (0.65–0.85)
Median age, years (IQR)	31 (24–39)	28 (23–35)	<0.001	<0.001	1.04^a^ (0.25–4.28)
Intercourse with a sex worker, n (%)	38 (20%)	62 (14%)	<0.001	<0.001	4.07 (0.98–16.82)
nPEP provided, n (%)	169 (87%)	338 (75%)	<0.001	<0.001	2.69 (2.37–3.06)
Stopping treatment before 28 days, n (%)	9 (5%)	61 (27%)	<0.001	<0.001	0.18 (0.08–0.39)
	**Condom use (n = 633)**		**Univariate analysis (p)**	**Multivariate analysis (p)**	**OR (95% CI)**
	**Yes (n = 348)**	**No (n = 285)**			
Multiple exposures during the study, n (%)	60 (17%)	21 (7%)	<0.001	0.007	3.54 (0.48–33.45)
Median delay between sexual exposure and nPEP evaluation at HIV infection care center, hours (IQR)	17 (7–38)	24 (10–47)	<0.001	0.004	0.91^b^ (0.76–1.09)
Intercourse with a sex worker, n (%)	85 (24%)	14 (5%)	<0.001	<0.001	10.36 (1.93–55.51)
Reported anal/vaginal ejaculation, n (%)	150 (43%)	162 (57%)	<0.001	0.015	0.38 (0.17–0.84)
Rape, n (%)	0	66 (23%)	<0.001	<0.001	2.10^−22^ (0-+∞)
Unprotected sexual intercourse during the last 6 months, n (%)	60 (17%)	72 (25%)	<0.001	<0.001	0.49 (0.24–0.99)

### Condom use

Reported condom use was 55% (n = 348). In the multivariate analysis (Table 2), a shorter time delay from sexual exposure to arrival in HIV-infection care center was associated with condom use (OR decrease per hour since exposure, 0.91 (0.76–1.09); p = 0.004). Besides, condom use increased with multiple exposures over the study period (OR, 3.54 [0.48–33.45]; p = 0.007). Reported condom use was strongly associated with a sexual intercourse with a sex worker (OR, 10.36 [1.93–55.51]; p<0.001). Decreased rates of condom use were found when patients reported unprotected sexual intercourse during the last six months (OR, 0.49 [0.24–0.99]; p<0.001), reported anal/vaginal ejaculation (OR, 0.38 [0.17–0.84]; p = 0.015) and in case of rape scenarios (OR, 2.10^−22^ [0-+∞]; p<0.001).

### nPEP prescription

Of 507 subjects who received nPEP, 460 (91%) were provided a short course NVP with zidovudine/lamivudine, 39 (8%) were protease inhibitor-based regimen and 8 (1%) were integrase inhibitor-based regimen. The decision of not using a NVP-short course was mostly based on resistance genotypes from the sexual partner and drug-drug interactions. Stopping nPEP regimen (Table 3) for adverse events was not different between the NVP-containing regimen group versus other regimen (p = 1).

**Table 3:** Reasons for stopping treatment according with nPEP regimen. AZT, zidovudine; 3TC, lamivudine; NVP, nevirapine.

	NVP (4 days) + AZT/3TC (28 days)	Other nPEP regimen	p value
	(n = 460)	(n = 47)	
Discontinuations for any reason, n (%)	**64 (14%)**	**6 (13%)**	1
Discontinuations for adverse events	21 (5%)	2 (4%)	1
Discontinuations due to lack of indication	21 (5%)	4 (9%)	0.3
Discontinuations for patients’ own reasons	15 (3%)	0	0.4
Discontinuations for other reasons	12 (3%)	0	0.6

Among patients with an evaluable 4-months ALT follow-up (n = 110), 20 of them showed increased ALT before nPEP initiation (grade 1, *n* = 17; grade 2, *n* = 3) and were not affected during follow-up. In 14 additional cases with normal rates at baseline, a moderate elevation was observed while treatment was ongoing at month 1 (grade 1, *n* = 13; grade 2, *n* = 1). Normalization occurred in 5 of these individuals at month 2, and the 9 last patients at the end of follow-up.

## Discussion

Sequential HIV-testing follow-up raises significant challenge to effective therapeutic intervention among patients exposed to HIV, including sexual encounter. Indeed, follow-up cares are necessary to reasonably exclude HIV infection related to the exposure and provide risk reduction counseling. Using five years of routinely gathered clinical data, we carried out an analysis of predictors of completing the recommended HIV-testing follow-up on 646 non-occupational exposures. Characteristics of the study population are similar to other recently nPEP users cohort [6–9], with no important trends observed. Predictors of condom use were also consistent with these cohorts. We found an HIV-testing FC of 30%, similarly to other studies as we used the same FC definition [17–18].

In global population, HIV-testing is associated to HIV risk perception [21–22]. We showed that sequential serologic-testing completion after sexual exposure to HIV is also linked to HIV risk perception. Indeed, FC decreased with age and the year of sexual exposure, which reflects a low level of HIV risk perception increasing over time, especially among young people [23]. In a context of post-exposure prophylaxis, changes in HIV representation and sexual behaviors have to be considered. This is consistent with studies in global population, which associate high-risk behaviors to an increased rate of having had an HIV test in the previous year [24]. Besides, an intercourse with a sex worker was linked to a better FC. This is supported by a high HIV-risk transmission perception among clients of sex workers. This risk perception is probably also due to stigma and irrational fear related to HIV infection, particularly on sex workers, or to the feeling of guilt which generally follows the access to sex workers [25].

Surprisingly, although, we could have expected that those who were given nPEP would be reassured by taking nPEP and subsequently be less likely to complete follow-up, we found that a complete HIV-testing follow-up was strongly associated with nPEP use. Conversely, patients who did not receive nPEP were less likely to complete follow-up. So, we suppose these patients were less worried to do so, because generally they did not receive nPEP as the referent physician did not estimate that it was recommended [21–22]. This could explain the different FC rates between nPEP and non-nPEP users. Although addressed in the national guidelines, lack of estimated risk could be considered not requiring monitoring. A lost of follow-up was also associated with an early discontinuation of the nPEP regimen. As reasons for not completing nPEP regimen were mostly adverse events, these patients were demotivated to complete their follow-up. Factors associated with. FC were different from those associated in nPEP regimen completion [14–15].

Almost all study patients took a short course of NVP regimen associated with two NRTIs. We acknowledge, this antiretroviral combination is currently not included in the national guidelines. Similarly to other recent nPEP studies [6–9], we found low rates of nPEP discontinuation due to adverse events (5%), although we acknowledged that lamivudine/zidovudine-based regimen have historically been associated with adverse events in several published reports [26].

Only 9% of partners with unknown status could be tested through the study. Improving HIV-testing of the sexual partner with the use of rapid HIV tests for low risk people or HIV RNA testing for high risk exposures could decrease nPEP toxicity and cost.

Although there were no documented nPEP failures, five MSM nPEP recipients subsequently seroconverted to HIV indicating ongoing risk behaviors. An elevated risk of subsequent seroconversion among nPEP recipients has recently been described [27]. Over the study period, we identified that seroconversion to HIV was significantly associated with the lack of HIV-testing completion among nPEP recipients. Multiple presentations for nPEP could be used to determine who might benefit from tailored multiple health promotion interventions, including HIV pre-exposure prophylaxis.

Our study may have several bias, as it is retrospective and mono-centric. We failed to quantify clinical toxicity rates as an important measure of nPEP success. Although standardized in medical records [28], missing values due to variation in provider practice (<10% by item), limited our study. Additionally, information regarding patient behavior, such as reported condom use, was determined through self-report and is subject to recall bias.

To the best of our knowledge, this is the first study that assessed predictors of completing HIV-testing follow-up in a context of non-occupational post-exposure prophylaxis. We showed that many individuals at risk of contracting HIV infection had not fulfilled sequential testing because they did not appear to recognize their HIV risk. As these individuals were mostly young adults recently exposed, understanding their underlying HIV risk perceptions changes is critical to implementing efficient prevention policies. We suggest that providers may require additional training in counseling, and how to tailor services to patients’ needs. Targeted individuals with high risk behaviors with enhanced counseling may be interesting [29]. Mobile phone or internet-based strategies might also enhance follow-up rates [30]. Indeed, some tools like eHealth, mHealth and “Web 2.0” social media strategies have demonstrated efficacy to reach and engage key populations in testing and care continuum.

Our findings highlight the need to consider segmented health promotion services for counseling regarding safer sexual behaviors as an integral component of nPEP care.
